# Evaluating the Impact of Electronic Prescribing on Medication Administration

**DOI:** 10.1192/bjo.2025.10633

**Published:** 2025-06-20

**Authors:** Monira Miah, Ivan Shanley, Stephanie Ko, Vanessa Jacobs

**Affiliations:** Rochford Community Hospital, Southend-on-Sea, United Kingdom

## Abstract

**Aims:** With the introduction of electronic prescribing (EP) to an older adult inpatient psychiatric ward after many years of paper charts, it was anticipated that unfamiliarity with the system would disrupt medication administration. This study sought to quantify that. The measure of disruption was defined as the deviation between the time of prescribed administration and the time medication was actually given.

**Methods:** A sample of ten patients was analysed across four dates. The first day of using EP was explored, followed by the final weekday of this week. The remaining dates were at week three and five of use. Data collected included the total number of drugs, doses and any deviation in the time administered from the time in the prescription (a delayed administration was recorded as a positive figure, and a premature administration was recorded as a negative figure, both in minutes). Using the time taken per dose and number of doses, the average time taken per patient was calculated for each date. This considered any changes made to treatment regimes, focussing primarily on timing of medication administration per dose.

**Results:** On the first day of using EP, a mean deviation of +10.6-minutes was seen across all patients (i.e. delay). By the end of week one, this dropped to a −11.5-minute deviation from the prescribed time (i.e. administered earlier). At week three, the mean was −9.7-minutes. By week five that fell to −5.3-minutes. The first day of using EP showed the longest mean delay seen for a single patient at 28.2 minutes. This dropped to 15 minutes by the end of the week, and further to 13.1 minutes at week three. The highest mean delay in a single patient however increased to 21.7 minutes by week five. In terms of individual doses, the number administered earlier than their prescribed time was lowest with initial use of EP, at 32 doses. By the end of the week, this more than doubled (75). The increased number of premature administrations dropped slightly in week three to 71 doses. At week five, it further fell to 66 doses.

**Conclusion:** There is no consistent evidence to suggest that the introduction of EP produces a sustained impact on the administration of medications. An understandable impact was noted on day one, however subsequent dates do not suggest any ongoing pattern. There is also no evidence to suggest that continued use improved adherence to prescribed times thereafter, with significant variability persisting.

